# Weight Regain Trajectories After Discontinuation of Semaglutide or Tirzepatide: A Reconstructed Aggregate‐Data Bayesian Longitudinal Meta‐Analysis

**DOI:** 10.1002/edm2.70325

**Published:** 2026-08-31

**Authors:** Chia Siang Kow, Kaeshaelya Thiruchelvam, Dinesh Sangarran Ramachandram, Abdullah Faiz Zaihan

**Affiliations:** ^1^ School of Pharmacy, IMU University Kuala Lumpur Malaysia; ^2^ School of Applied Sciences, University of Huddersfield Huddersfield UK; ^3^ School of Pharmacy, Monash University Subang Jaya Selangor Malaysia; ^4^ Department of Pharmacy Shah Alam Hospital Shah Alam Selangor Malaysia

**Keywords:** Bayesian hierarchical model, Bayesian meta‐analysis, incretin‐based therapy, obesity, semaglutide, tirzepatide, treatment discontinuation, weight regain

## Abstract

**Background:**

Semaglutide and tirzepatide produce substantial weight loss during treatment, but the trajectory of weight regain after discontinuation remains uncertain.

**Objective:**

To estimate post‐discontinuation weight‐regain trajectories and explore predictors of faster regain.

**Methods:**

We used published timepoint‐level aggregate data from studies evaluating weight change after discontinuation of semaglutide or tirzepatide to perform a Bayesian hierarchical longitudinal re‐analysis. The model estimated weight loss at cessation, monthly regain rate, time to 50% regain, and time to return to baseline weight. Bayesian meta‐regression examined medication type, magnitude of initial weight loss, and post‐discontinuation behavioural or lifestyle support. Longer‐term estimates assumed a constant linear regain rate.

**Results:**

Six studies comprising 10 intervention arms and 1776 participants were included, with observed follow‐up ranging from 4 to 52 weeks. Estimated weight loss at cessation was 15.35 kg (95% credible interval [CrI] 11.18–19.60), followed by regain of 1.04 kg/month (95% CrI 0.80–1.29). Based on the linear model, 50% of initial weight loss was projected to be regained by 7.50 months (95% CrI 5.05–10.57), with return to baseline weight by 15.00 months (95% CrI 10.10–21.13). Tirzepatide showed numerically faster regain than semaglutide in unadjusted analyses, but no clear independent drug‐specific difference was evident after adjustment. Greater initial weight loss showed the strongest directional association with faster regain, although the credible interval included zero. Behavioural or lifestyle support was directionally associated with slower regain, but the estimate was imprecise.

**Conclusions:**

Weight regain after discontinuation of semaglutide or tirzepatide was rapid and clinically meaningful. Because estimates beyond 52 weeks were model‐based extrapolations, they should be interpreted cautiously. Early monitoring and proactive maintenance planning may be warranted after treatment cessation.

## Graphical abstract

After discontinuation of semaglutide or tirzepatide, weight regain averaged 1.04 kg/month. Based on a linear model, half of treatment‐associated weight loss was projected to return by approximately 7.5 months and baseline weight by approximately 15 months, highlighting the need for early monitoring and maintenance planning.

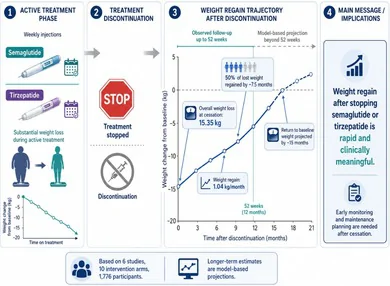

## 1 Introduction

Obesity is a chronic and relapsing condition associated with substantial cardiometabolic, functional, and healthcare burden [1, 2, 3]. The emergence of newer incretin‐based anti‐obesity pharmacotherapies, particularly semaglutide and tirzepatide, has changed the therapeutic landscape by producing clinically meaningful weight reductions that exceed those historically achieved with lifestyle intervention alone or older anti‐obesity medications. These therapies are increasingly used not only for weight reduction but also as part of broader cardiometabolic risk management [4, 5].

However, the long‐term value of anti‐obesity pharmacotherapy depends not only on the magnitude of weight loss achieved during active treatment, but also on whether these benefits are sustained after treatment discontinuation. This is especially important because discontinuation is common in routine practice due to cost, tolerability, access restrictions, patient preference, treatment fatigue, and uncertainty regarding the optimal duration of therapy [6, 7]. As these agents act partly by reducing appetite and improving satiety during active treatment, withdrawal of therapy may remove the physiological support that enabled weight loss, resulting in progressive weight regain [7, 8].

A recent systematic review and meta‐analysis [9] provided the most comprehensive evidence to date on weight regain after cessation of weight‐management medications. That review included 37 studies, 63 intervention arms and 9341 participants. Across all medications, the average rate of weight regain was 0.4 kg/month, with projected return to baseline weight by 1.7 years. Regain was faster after newer incretin‐based therapies, including semaglutide and tirzepatide, with an estimated regain rate of 0.8 kg/month and projected return to baseline weight by approximately 1.5 years. The six studies and 10 semaglutide or tirzepatide arms analysed here were drawn entirely from that source review, so study overlap was complete by design.

Although these findings are clinically important, average monthly regain estimates alone may not fully address the questions most relevant to clinical decision‐making. Clinicians and patients may need to know not only how many kilograms are regained per month, but also when clinically meaningful regain is likely to occur. For example, the time to regain 50% of initial weight loss, the time to return to baseline weight, and the probability of substantial regain by 6, 9, or 12 months may be more directly useful for counselling, monitoring and planning maintenance strategies. Similarly, apparent differences between semaglutide and tirzepatide may reflect differences in the magnitude of initial weight loss rather than true drug‐specific differences in post‐discontinuation rebound.

A Bayesian hierarchical longitudinal approach can address these gaps by jointly modelling repeated timepoint‐level aggregate data and between‐arm heterogeneity. In the present analysis, post‐discontinuation weight regain was represented by a constant linear arm‐specific slope rather than a nonlinear curve; this assumption was selected because follow‐up was sparse and the source review found that adding a curvilinear term did not improve model fit [9]. The Bayesian framework provides posterior distributions for clinically interpretable average‐trajectory estimates, including time to 50% regain and probability of returning to baseline weight, while meta‐regression can explore whether medication type, initial weight loss, and post‐discontinuation support are associated with the regain slope.

Therefore, this study aimed to perform a reconstructed aggregate‐data Bayesian re‐analysis of the semaglutide and tirzepatide studies included in the source review. The primary objective was to estimate the average post‐discontinuation weight‐regain trajectory using a Bayesian hierarchical longitudinal model and to translate the constant linear regain rate into clinically interpretable milestones. The secondary objective was to explore medication type, magnitude of initial weight loss, and post‐discontinuation behavioural or lifestyle support as predictors of the regain slope.

## 2 Methods

### 2.1 Study Design and Data Source

This study was designed as a reconstructed aggregate‐data Bayesian re‐analysis of published evidence [9] on weight regain after discontinuation of newer incretin‐based pharmacotherapy. The analysis used the complete subgroup of semaglutide and tirzepatide studies and intervention arms reported in the source systematic review and its Supporting Information; therefore, there was no additional study selection beyond this prespecified pharmacological restriction.

No updated systematic literature search was performed. The source review searched trial registries and bibliographic databases from inception through February 2025 [9]. Eligible studies, intervention characteristics, treatment durations, follow‐up durations, and post‐discontinuation support strategies were identified from that review and its Supporting Information.

### 2.2 Eligibility Criteria

Studies were eligible if they contributed post‐discontinuation weight‐change data for semaglutide or tirzepatide among adults with overweight or obesity. The primary focus was use for weight management. SURPASS‐1 was retained because participants had a mean baseline body mass index of 32.3 kg/m^2^ and the publication provided dose‐specific weight‐change data during a defined off‐treatment period. However, it enrolled adults with Type 2 diabetes and evaluated tirzepatide as glucose‐lowering monotherapy; therefore, a post hoc sensitivity analysis excluded its three intervention arms. Randomised controlled trials and eligible trial arms with at least one post‐discontinuation assessment were included. Excluded therapies were grouped by pharmacological class and comprised other incretin‐based agents such as liraglutide; the long‐acting amylin analogue cagrilintide; the lipase inhibitor orlistat; sympathomimetic or other centrally acting agents, including phentermine, sibutramine, lorcaserin and fenfluramine; the cannabinoid‐1 receptor antagonist rimonabant; topiramate; and other non‐semaglutide or non‐tirzepatide therapies.

### 2.3 Data Extraction and Reconstruction

Arm‐level study characteristics were extracted, including study name, year, drug, dose, number of participants, treatment duration, follow‐up duration, and type of post‐discontinuation support. One author manually transcribed timepoint‐level weight‐change estimates from rendered supplementary forest plots with optical character recognition assistance. A second author checked the sample sizes, means, and confidence limits against the rendered source plots, and discrepancies were resolved before analysis. Where available, randomised controlled trial contrast data were also extracted as the difference in weight change from baseline between intervention and control arms.

For each timepoint, the extracted variables included study arm, drug, dose, post‐discontinuation follow‐up time, mean weight change from baseline, 95% confidence interval, and sample size. Standard errors were derived from reported 95% confidence intervals using the standard normal approximation. Follow‐up time was converted from weeks to months for modelling.

### 2.4 Outcomes

The primary outcome was the modelled average post‐discontinuation weight‐regain trajectory, expressed as change in body weight from baseline over time after stopping semaglutide or tirzepatide. Threshold‐crossing probabilities described uncertainty in the modelled average trajectory and were not individual‐patient probabilities. The main model‐derived outcomes were:

1. monthly rate of weight regain;
2. estimated time to regain 50% of the initial treatment‐associated weight loss;
3. estimated time to return to baseline weight;
4. posterior probability of regaining at least 50% of initial weight loss at selected time points;
5. posterior probability of returning to baseline weight at selected time points

The secondary outcome was identification of predictors of faster post‐discontinuation weight regain.

### 2.5 Primary Bayesian Hierarchical Longitudinal Model

The primary analysis used a Bayesian hierarchical longitudinal model to estimate weight‐regain trajectories after treatment discontinuation. The model specified a constant linear rate of weight regain after cessation. Each study arm was treated as a repeated‐measures trajectory, with arm‐level random effects for treatment‐associated weight loss at cessation and the subsequent regain slope to account for between‐arm heterogeneity.

The model estimated the amount of weight loss present at treatment discontinuation and the subsequent monthly regain slope. Drug‐specific estimates were generated for semaglutide and tirzepatide, together with an overall pooled estimate for newer incretin‐based therapies.

Time to 50% regain was calculated as half of the estimated initial weight loss divided by the estimated constant monthly regain slope. Time to return to baseline was calculated as the estimated initial weight loss divided by the same slope. Posterior distributions were used to derive medians and 95% credible intervals. Estimates extending beyond the maximum observed follow‐up of 52 weeks were explicitly treated as linear model‐based extrapolations of the average trajectory.

### 2.6 Secondary Bayesian Meta‐Regression

A secondary Bayesian meta‐regression was performed to explore predictors of faster post‐discontinuation weight regain. The dependent variable was the arm‐specific monthly regain slope. Candidate predictors included medication type, magnitude of initial treatment‐associated weight loss, and post‐discontinuation behavioural or lifestyle support.

Medication type was coded as tirzepatide versus semaglutide. Initial weight loss was modelled per 5 kg greater weight loss at discontinuation. Post‐discontinuation support was coded as behavioural or lifestyle support versus no active support. Results were expressed as regression coefficients in kg/month, with 95% credible intervals and posterior probabilities for the direction of effect.

### 2.7 Sensitivity Analysis

Two sensitivity analyses were undertaken. First, randomised controlled trial contrast data were analysed using the difference in weight change from baseline between intervention and control arms after treatment discontinuation. Second, in response to the difference in population and treatment indication, the primary absolute‐change model was repeated after excluding the three SURPASS‐1 intervention arms. This post hoc analysis assessed whether inclusion of adults with Type 2 diabetes receiving tirzepatide monotherapy materially affected the findings.

### 2.8 Model Estimation and Diagnostics

Models were estimated using full Markov chain Monte Carlo (MCMC) sampling. Posterior summaries were reported as medians with 95% credible intervals. Model convergence was assessed using R‐hat statistics, effective sample sizes, and visual inspection of posterior sampling behaviour. R‐hat values close to 1.00 were considered consistent with adequate convergence.

## 3 Results

### 3.1 Study Characteristics of the Analysed Subgroup

The Bayesian re‐analysis included six studies evaluating semaglutide or tirzepatide after treatment discontinuation [10, 11, 12, 13, 14, 15]. These comprised 10 intervention arms and 1776 participants. The semaglutide subgroup included three studies [12, 14, 15], three intervention arms, and 634 participants, while the tirzepatide subgroup included three studies [10, 11, 13], seven intervention arms, and 1142 participants. Observed post‐discontinuation follow‐up ranged from 4 to 52 weeks, contributing 53 timepoint‐level observations across the primary and control‐contrast datasets (Table 1).

**TABLE 1:** Characteristics of studies included in the Bayesian re‐analysis.

Study	Design	Population	Drug and dose	Participants analysed, *n*	Age, years	Female, *n* (%)	Baseline weight, kg	Baseline BMI, kg/m^2^	Glycaemic status	Treatment duration	Post‐discontinuation follow‐up	Post‐discontinuation support	Risk of bias
Aronne et al. 2023/SURMOUNT‐4 [10]	Randomised withdrawal trial	Adults with obesity or overweight without diabetes	Tirzepatide 10 or 15 mg once weekly	335	48	237 (70.7)	107.3 at treatment start; 85.8 at discontinuation	38.4 at treatment start; 30.7 at discontinuation	No diabetes	36 weeks	52 weeks	Switched to placebo; lifestyle counselling continued	Low
Jastreboff et al./SURMOUNT‐1 extension [11]	Randomised controlled trial extension	Adults with obesity or overweight and prediabetes	Tirzepatide 5, 10, or 15 mg once weekly	541	48.3	64.2	107.4	38.7	Prediabetes; diabetes excluded	176 weeks	17 weeks	Off‐treatment follow‐up after tirzepatide cessation	Some concerns
McGowan et al. 2024/STEP 10 [12]	Randomised, double‐blind, placebo‐controlled trial	Adults with obesity and prediabetes	Semaglutide 2.4 mg once weekly	138	53 (11)	100 (72.0)	111.9 (21.5)	39.9 (6.6)	Prediabetes; diabetes excluded	52 weeks	28 weeks	Healthy lifestyle counselling during off‐treatment period	Low
Rosenstock et al. 2023/SURPASS‐1 follow‐up [13]	Off‐treatment follow‐up of randomised trial	Adults with Type 2 diabetes receiving tirzepatide monotherapy	Tirzepatide 5, 10, or 15 mg once weekly	266	54.6	122 (45.9)	87.4	32.3	Type 2 diabetes	40 weeks	4 weeks	No study treatment or glucose‐lowering therapy during follow‐up	Some concerns
Rubino et al. 2021/STEP 4 [14]	Randomised withdrawal trial	Adults with obesity or overweight without diabetes	Semaglutide 2.4 mg once weekly	268	46 (12)	205 (76.5)	107.2 at treatment start; 95.4 at discontinuation	38.4 at treatment start; 34.1 at discontinuation	No diabetes	20 weeks	48 weeks	Switched to placebo; lifestyle intervention continued	Low
Wilding et al. 2022/STEP 1 extension [15]	Off‐treatment extension of randomised trial	Adults with obesity or overweight without diabetes	Semaglutide 2.4 mg once weekly	228	48 (12)	152 (66.7)	105.6 (21.8) at treatment start; 87.5 (21.4) at discontinuation	37.6 (7.0) at treatment start; 31.2 (7.2) at discontinuation	No diabetes; prediabetes in 142 (62.3%)	68 weeks	52 weeks	Treatment and active lifestyle intervention discontinued	Low

### 3.2 Primary Bayesian Hierarchical Longitudinal Model

In the primary full MCMC Bayesian hierarchical longitudinal model, the overall estimated treatment‐associated weight loss at cessation was 15.35 kg (95% credible interval [CrI] 11.18–19.60 kg), and the constant monthly regain slope was 1.04 kg/month (95% CrI 0.80–1.29 kg/month). Under this linear assumption, 50% of the initial weight loss was projected to be regained by 7.50 months (95% CrI 5.05–10.57 months), and return to baseline was projected by 15.00 months (95% CrI 10.10–21.13 months) (Table 2). The return‐to‐baseline estimate extends beyond the maximum observed follow‐up of 52 weeks and is therefore an extrapolation rather than an observed outcome.

**TABLE 2:** Bayesian hierarchical longitudinal model and Bayesian meta‐regression of weight regain after discontinuation of newer incretin‐based anti‐obesity pharmacotherapy.

Parameter	Overall	Semaglutide	Tirzepatide
Panel A. Bayesian hierarchical longitudinal model			
Weight loss at treatment discontinuation, kg	15.35 (11.18–19.60)	14.80 (7.32–22.06)	15.59 (10.46–20.74)
Monthly weight‐regain slope, kg/month	1.04 (0.80–1.29)	0.89 (0.56–1.23)	1.10 (0.79–1.43)
Time to 50% regain, months	7.50 (5.05–10.57)	8.65 (3.93–15.24)	7.24 (4.43–10.97)
Time to return to baseline weight, months	15.00 (10.10–21.13)	17.30 (7.85–30.47)	14.48 (8.86–21.94)
Between‐arm SD for weight loss at discontinuation, kg	6.78 (4.29–11.06)	—	—
Between‐arm SD for monthly regain slope, kg/month	0.26 (0.12–0.51)	—	—

Predictor	Regression coefficient, kg/month	95% credible interval	Posterior probability for direction of effect
Panel B. Bayesian meta‐regression for monthly weight‐regain slope			
Intercept: semaglutide, no post‐discontinuation support, at mean initial weight loss	1.01	0.70 to 1.36	—
Tirzepatide versus semaglutide	−0.04	−0.46 to 0.44	40.2% probability of faster regain
Initial weight loss, per 5 kg greater loss	0.20	−0.10 to 0.44	92.1% probability of faster regain
Behavioural/lifestyle support after discontinuation	−0.19	−0.57 to 0.17	87.2% probability of slower regain
Residual between‐arm SD	0.17	0.03 to 0.42	—

*Note:* Observed post‐discontinuation follow‐up extended to 52 weeks (approximately 12 months). Time‐to‐threshold estimates extending beyond 12 months are extrapolations from the constant linear modelled average trajectory.

Drug‐specific monthly regain slopes were 0.89 kg/month (95% CrI 0.56–1.23) for semaglutide and 1.10 kg/month (95% CrI 0.79–1.43) for tirzepatide. The corresponding projected times to 50% regain were 8.65 months (95% CrI 3.93–15.24) and 7.24 months (95% CrI 4.43–10.97), while projected return to baseline was 17.30 months (95% CrI 7.85–30.47) and 14.48 months (95% CrI 8.86–21.94), respectively (Table 2). These threshold estimates are model‐derived and the return‐to‐baseline estimates extend beyond observed follow‐up.

Between‐arm heterogeneity was present for both initial weight loss and post‐discontinuation regain. The estimated between‐arm standard deviation for initial weight loss was 6.78 kg (95% CrI 4.29–11.06 kg), while the between‐arm standard deviation for regain slope was 0.26 kg/month (95% CrI 0.12–0.51 kg/month) (Table 2).

### 3.3 Modelled Weight‐Regain Trajectory

**FIGURE 1:**
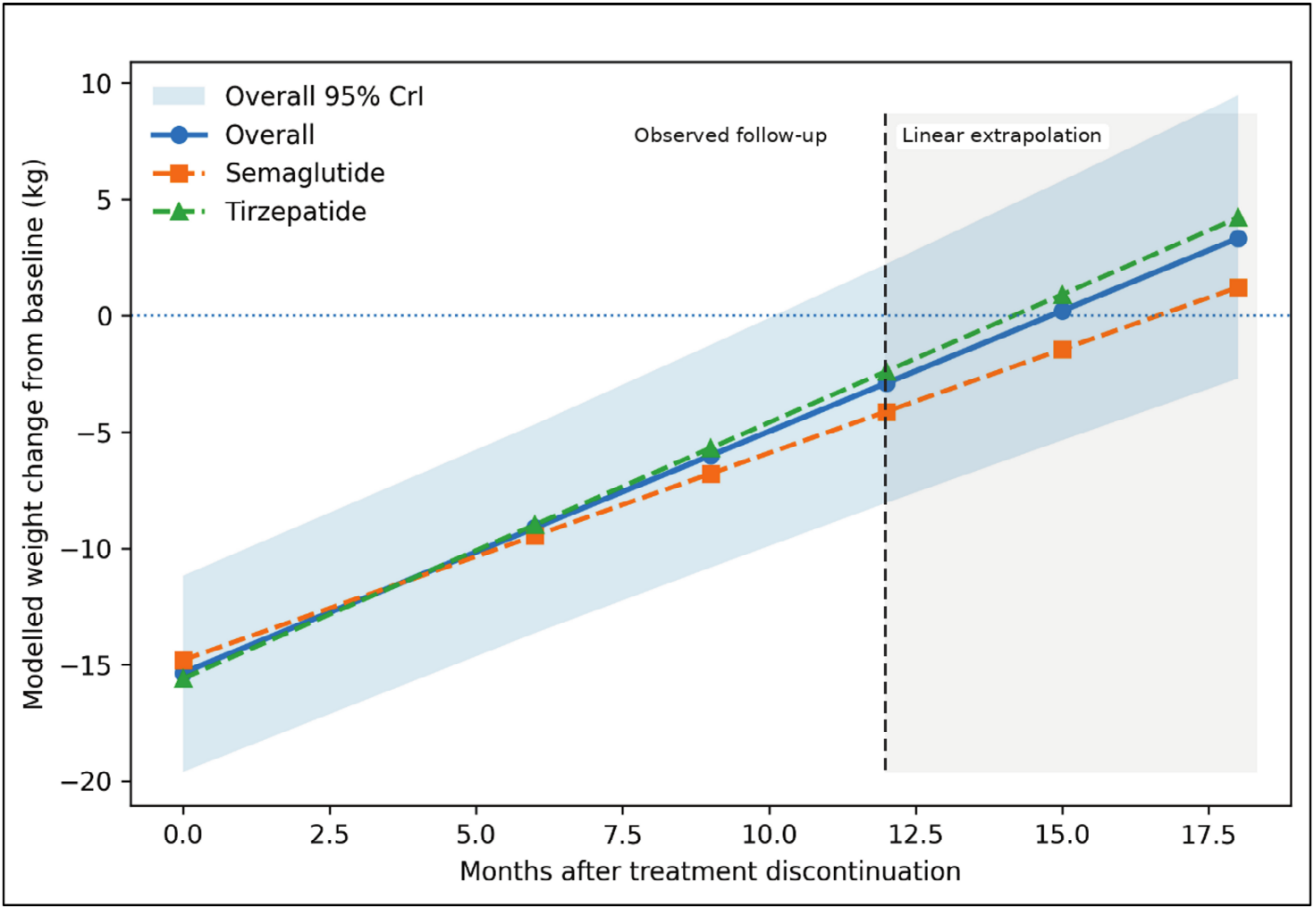
Modelled weight‐regain trajectory after discontinuation of semaglutide or tirzepatide. The solid line shows the overall posterior median modelled weight change from baseline, with shaded 95% credible intervals. Dashed lines show drug‐specific median trajectories for semaglutide and tirzepatide. The horizontal reference line represents return to baseline weight. The vertical dashed line marks the maximum observed follow‐up of 52 weeks (approximately 12 months); the region to its right represents linear model‐based extrapolation.

Within the observed follow‐up window, the modelled average weight change remained below baseline at 6 months (−9.12 kg; 95% CrI −13.64 to −4.68), 9 months (−6.01 kg; 95% CrI −10.82 to −1.26) and 12 months (−2.90 kg; 95% CrI −8.04 to 2.22). These estimates corresponded to approximately 41.4%, 62.1% and 82.8% of initial weight loss regained, respectively (Figure 1).

At 15 and 18 months, which were beyond the maximum observed follow‐up, the constant linear model projected mean weight changes of 0.21 kg (95% CrI −5.35 to 5.80) and 3.33 kg (95% CrI −2.70 to 9.46) relative to baseline. These values are extrapolations of the modelled average trajectory and should not be interpreted as directly observed outcomes (Figure 1).

### 3.4 Probability of Clinically Important Regain

**FIGURE 2:**
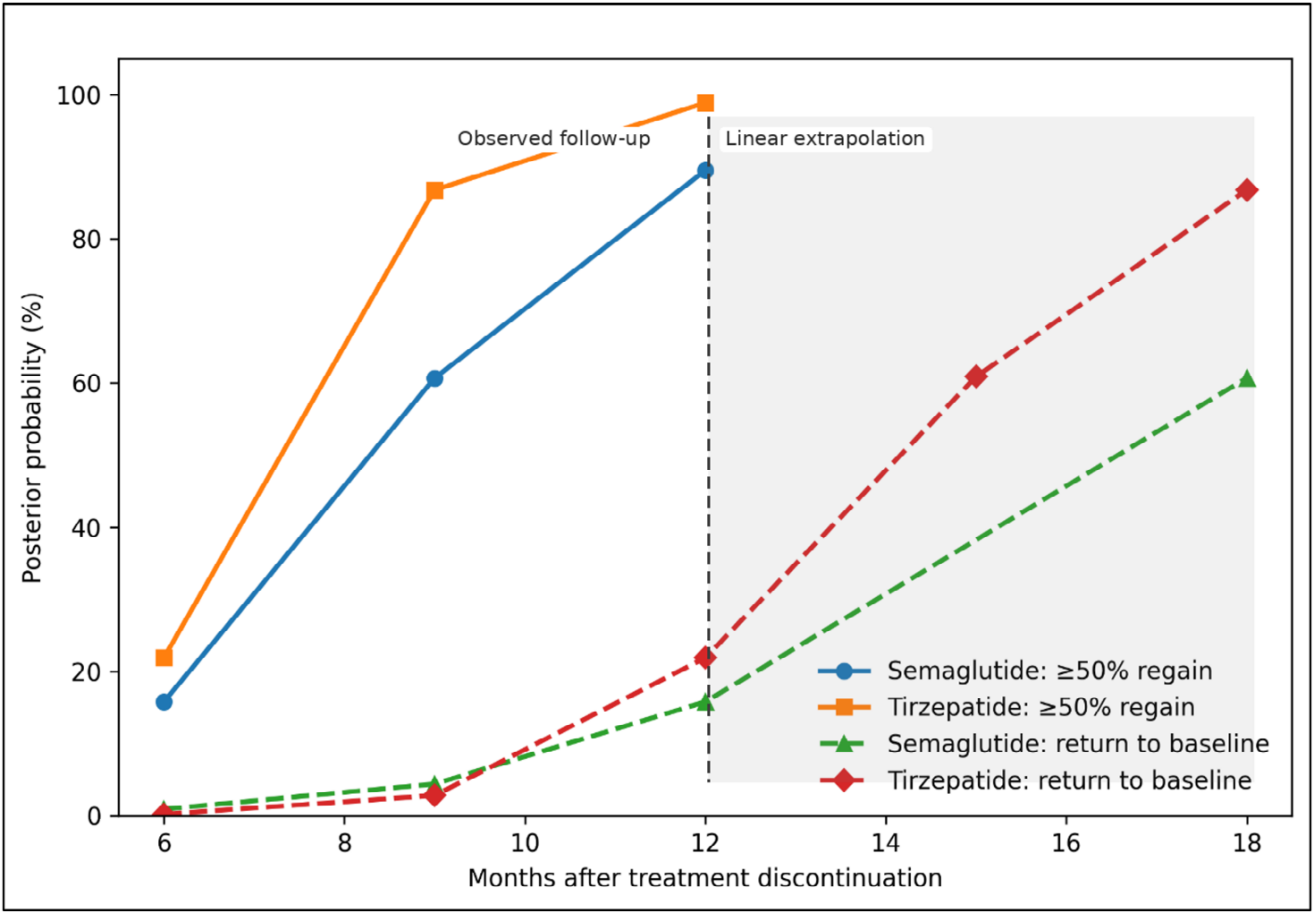
Posterior probability of clinically important weight regain after discontinuation. The figure shows the posterior probability that the modelled average trajectory had regained at least 50% of initial treatment‐associated weight loss or returned to baseline weight at selected timepoints. These are average‐trajectory probabilities, not individual‐patient probabilities. The vertical dashed line marks the maximum observed follow‐up of 52 weeks (approximately 12 months); probabilities beyond this point are linear model‐based extrapolations.

Across all arms, the posterior probability that the modelled average trajectory had regained at least 50% of initial weight loss was 12.5% at 6 months, 86.7% at 9 months, and 99.5% at 12 months. The corresponding average‐trajectory probability of return to baseline was 0.03% at 6 months, 0.8% at 9 months and 12.5% at 12 months. Probabilities at 15 and 18 months were extrapolated beyond observed follow‐up and are displayed separately in Figure 2.

Drug‐specific posterior probabilities are presented in Figure 2. They describe uncertainty regarding each drug subgroup's modelled average trajectory and do not represent the probability that an individual patient will experience the specified degree of regain.

### 3.5 Secondary Bayesian Meta‐Regression

Bayesian meta‐regression explored medication type, magnitude of initial weight loss, and behavioural or lifestyle support after treatment cessation as predictors of the monthly regain slope. Because only 10 intervention arms were available, all predictor analyses were considered exploratory.

In the adjusted model, the intercept for semaglutide arms at the mean level of initial weight loss and without post‐discontinuation support was 1.01 kg/month (95% CrI 0.70–1.36 kg/month). After adjustment, there was no clear independent difference between tirzepatide and semaglutide. Tirzepatide was associated with an adjusted effect of −0.04 kg/month (95% CrI −0.46 to 0.44 kg/month) on the regain slope compared with semaglutide. The posterior probability that tirzepatide was associated with faster regain was 40.2%, while the probability that it was associated with slower regain was 59.8% (Table 2).

Greater initial weight loss showed the strongest directional association with faster absolute regain. Each additional 5 kg of treatment‐associated weight loss was associated with a 0.20 kg/month faster regain slope (95% CrI −0.10 to 0.44 kg/month), with a 92.1% posterior probability of a positive association (Table 2). The credible interval included zero, and the estimate may partly reflect mathematical coupling or greater opportunity to regain after a larger initial loss.

Behavioural or lifestyle support after treatment cessation was directionally associated with slower regain. Support was associated with an estimated −0.19 kg/month effect on the regain slope (95% CrI −0.57 to 0.17 kg/month), with an 87.2% posterior probability of slower regain (Table 2). The credible interval included zero and the finding was inconclusive.

The estimated residual between‐arm standard deviation in the meta‐regression model was 0.17 kg/month (95% CrI 0.03–0.42 kg/month), suggesting remaining heterogeneity not fully explained by medication type, initial weight loss, or post‐discontinuation support (Table 2).

### 3.6 Sensitivity Analyses

The randomised controlled trial contrast analysis showed a similar direction of effect but was less precise because fewer control‐adjusted timepoints were available (Table S1). In the post hoc sensitivity analysis excluding the three SURPASS‐1 arms, the estimated monthly regain slope remained similar at 0.95 kg/month (95% CrI 0.69–1.23). Because the remaining arms had greater average weight loss at cessation, the projected time to 50% regain was longer at 9.42 months (95% CrI 7.03–13.26), and projected return to baseline was 18.83 months (95% CrI 14.07–26.52). Thus, exclusion of SURPASS‐1 did not change the conclusion that regain was rapid, but it affected threshold timing through the change in average initial weight loss.

### 3.7 Model Convergence

Full MCMC diagnostics supported adequate model convergence. For the primary model, R‐hat values were approximately 1.00, and effective sample sizes were high for the main slope parameters. The meta‐regression model also showed acceptable convergence, with R‐hat values close to 1.00 for all predictors (Table S2).

## 4 Discussion

In this reconstructed aggregate‐data Bayesian analysis, semaglutide or tirzepatide discontinuation was followed by an estimated constant regain rate of 1.04 kg/month. Approximately half of the initial treatment‐associated weight loss was projected to be regained by 7.5 months. However, observed follow‐up ended at 52 weeks; therefore, the projected return to baseline at approximately 15 months depends on continuation of the linear average regain rate beyond the available data.

The findings are consistent with the source systematic review, which estimated a regain rate of 0.8 kg/month and return to baseline at approximately 1.5 years for newer incretin‐based therapies [9]. Study overlap was complete because the present analysis reused the same six studies and 10 semaglutide or tirzepatide arms. The added value of the Bayesian re‐analysis is not identification of additional evidence, but joint modelling of repeated arm‐level timepoints, explicit propagation of uncertainty, and translation of the average slope into posterior distributions for clinically interpretable trajectory milestones.

Tirzepatide arms showed a numerically faster unadjusted regain slope than semaglutide arms, but adjusted meta‐regression did not support a clear independent drug‐specific difference. This comparison is limited by the small number of arms, differences in initial treatment‐associated weight loss, and heterogeneous follow‐up. Current evidence therefore does not establish that either drug has an inherently faster post‐discontinuation rebound pattern.

Greater initial weight loss showed the strongest directional association with faster absolute regain, but this exploratory result requires caution. The analysis included only 10 intervention arms, the 95% credible interval included zero, and larger initial loss creates greater opportunity for absolute regain. Mathematical coupling between initial loss and the subsequent absolute slope may also contribute. The finding should therefore be regarded as hypothesis‐generating rather than evidence of an independent prognostic factor.

Behavioural or lifestyle support was directionally associated with a slower regain slope, although the estimate was imprecise and crossed zero. Support strategies differed substantially across studies and were generally not randomised specifically as maintenance interventions. This result does not establish effectiveness, but it supports direct trials of structured post‐discontinuation support and standardised reporting of its intensity, duration, and adherence.

The model may inform the timing of clinical surveillance after treatment cessation. Monitoring within the first several months could identify early regain and prompt reassessment of weight‐management goals and available maintenance options. These are clinical implications rather than interventions tested by the present analysis; the data do not determine whether tapering, lower‐dose maintenance, switching, intermittent treatment, or any specific behavioural programme prevents regain.

Potential reversal of cardiometabolic benefits, cost‐effectiveness, reimbursement design, and the consequences of fixed‐duration treatment policies are important broader considerations [7, 16, 17]. Nevertheless, the present model assessed body‐weight trajectories only. Statements about cardiometabolic outcomes, economic value, or policy effects should therefore be interpreted as hypotheses informed by the weight‐regain pattern and existing literature, not as direct findings of this analysis.

SURPASS‐1 differed from the other studies because it enrolled adults with Type 2 diabetes and evaluated tirzepatide as glucose‐lowering monotherapy. Its inclusion was justified by the participants' mean BMI in the obesity range and the availability of dose‐specific off‐treatment weight data. Excluding its three lower‐weight‐loss arms produced a similar monthly regain slope but longer projected threshold times because average weight loss at cessation increased. This sensitivity analysis supports the qualitative conclusion while highlighting that glycaemic status, indication, and initial weight loss affect generalisability and model‐derived milestones.

Future studies should extend observed follow‐up beyond 12 months, distinguish observed estimates from extrapolations, and report percentage of lost weight regained alongside absolute change. Trials should directly compare discontinuation and maintenance strategies, while individual‐participant‐data analyses should examine diabetes status, baseline body mass index, sex, age, appetite, activity, adherence, and socioeconomic factors. Flexible nonlinear models will become more informative when sufficient longer‐term timepoints are available.

The main strength of this analysis is its clinically focused Bayesian longitudinal framework, which jointly modelled repeated aggregate timepoints and expressed uncertainty probabilistically. Limitations include reliance on reconstructed aggregate rather than individual‐level data, only six studies and 10 arms, heterogeneous post‐discontinuation support, and a linear slope assumption. The meta‐regression was underpowered and exploratory. Most importantly, projections after 52 weeks are conditional on a constant average regain rate and should not be interpreted as observed long‐term outcomes or individual‐patient predictions.

## 5 Conclusion

In this reconstructed aggregate‐data Bayesian re‐analysis, discontinuation of semaglutide or tirzepatide was associated with rapid weight regain. Under a constant linear average‐trajectory model, approximately half of the initial treatment‐associated weight loss was projected to return within 7–9 months. Projected return to baseline occurred after approximately 14–17 months, but this estimate extends beyond the maximum observed follow‐up of 52 weeks and should be interpreted as extrapolation. Greater initial weight loss showed the strongest directional association with faster absolute regain, although the credible interval included zero and the analysis was exploratory. Adjusted drug‐specific differences and the apparent benefit of behavioural or lifestyle support remained uncertain. These findings support proactive planning and early follow‐up when newer incretin‐based anti‐obesity therapy is discontinued.

## Author Contributions

**Dinesh Sangarran Ramachandram:** writing – review and editing, methodology, formal analysis. **Abdullah Faiz Zaihan:** writing – review and editing, conceptualization, supervision, methodology, project administration. **Kaeshaelya Thiruchelvam:** methodology, validation, writing – review and editing. **Chia Siang Kow:** methodology, conceptualization, investigation, formal analysis, writing – original draft, data curation.

## Funding

The authors have nothing to report.

## Ethics Statement

The authors have nothing to report.

## Consent

The authors have nothing to report.

## Conflicts of Interest

The authors declare no conflicts of interest.

## Supporting information


**Table S1:** Sensitivity analysis using randomised controlled trial contrast data.
**Table S2:** Markov chain Monte Carlo convergence diagnostics.

## Data Availability

The data that support the findings of this study are available from the corresponding author upon reasonable request.
