# Supplementary material for: Weight Regain Trajectories After Discontinuation of Semaglutide or Tirzepatide: A Reconstructed Aggregate‐Data Bayesian Longitudinal Meta‐Analysis
Source: Endocrinol Diabetes Metab. 2026 Aug 31;9(5):e70325. doi: 10.1002/edm2.70325 (PMC13529306; doi:10.1002/edm2.70325)
Supplement: Supplementary file 1 [file EDM2-9-e70325-s001.docx]

**Supplementary Table S1. Sensitivity analysis using randomised controlled trial contrast data**

| Analysis | Estimate | 95% CrI | Posterior probability |
| --- | --- | --- | --- |
| Panel A. Bayesian longitudinal model using RCT contrast data | | | |
| Overall weight loss at cessation, kg | 12.68 | 11.29 to 14.06 | — |
| Overall regain slope, kg/month | 0.75 | 0.18 to 1.32 | 99.5% probability of positive regain |
| Overall time to 50% regain, months | 11.22 | 4.72 to 30.90 | — |
| Overall time to return to baseline, months | 22.01 | 9.43 to 61.33 | — |
| Semaglutide weight loss at cessation, kg | 13.47 | 11.50 to 15.41 | — |
| Semaglutide regain slope, kg/month | 0.73 | 0.47 to 1.00 | 100.0% probability of positive regain |
| Semaglutide time to 50% regain, months | 9.52 | 6.50 to 14.59 | — |
| Semaglutide time to return to baseline, months | 19.04 | 13.01 to 29.18 | — |
| Tirzepatide weight loss at cessation, kg | 11.89 | 9.95 to 13.83 | — |
| Tirzepatide regain slope, kg/month | 0.77 | −0.28 to 1.82 | Directionally positive but imprecise |
| Tirzepatide time to 50% regain, months | 15.30 | 3.14 to 70.80 | — |
| Tirzepatide time to return to baseline, months | 27.18 | 6.27 to 130.95 | — |
| Panel B. Bayesian meta-regression using RCT contrast data | |  |  |
| Tirzepatide versus semaglutide, kg/month | 0.18 | −0.29 to 0.64 | 77.1% probability of faster regain |
| Initial weight loss, per 5 kg greater loss, kg/month | 0.23 | −0.12 to 0.58 | 90.2% probability of faster regain |
| Treatment duration, per 52 weeks longer, kg/month | 0.09 | −0.14 to 0.32 | 78.0% probability of faster regain |
| Behavioural/lifestyle support after discontinuation, kg/month | −0.30 | −0.73 to 0.13 | 91.2% probability of slower regain |

Values are posterior means with 95% credible intervals. Positive values indicate greater post-discontinuation weight regain. The RCT contrast analysis used differences in weight change from baseline between intervention and control arms and included fewer eligible arms and timepoint observations than the primary absolute-change model. Therefore, this analysis was considered supportive.

**Supplementary Table S2. Markov chain Monte Carlo convergence diagnostics**

| Model | Parameter | R-hat | Effective sample size |
| --- | --- | --- | --- |
| Primary Bayesian longitudinal model | Semaglutide weight loss at cessation | 1.001 | 21,969 |
| Primary Bayesian longitudinal model | Tirzepatide weight loss at cessation | 1.000 | 21,979 |
| Primary Bayesian longitudinal model | Between-arm SD for initial weight loss | 1.000 | 22,003 |
| Primary Bayesian longitudinal model | Semaglutide regain slope | 1.000 | 21,998 |
| Primary Bayesian longitudinal model | Tirzepatide regain slope | 1.000 | 22,002 |
| Primary Bayesian longitudinal model | Between-arm SD for regain slope | 1.000 | 21,998 |
| Bayesian meta-regression | Intercept | 1.000 | 17,987 |
| Bayesian meta-regression | Tirzepatide versus semaglutide | 1.001 | 17,968 |
| Bayesian meta-regression | Initial weight loss, per 5 kg greater loss | 1.001 | 17,972 |
| Bayesian meta-regression | Behavioural/lifestyle support after discontinuation | 1.000 | 17,991 |
| Bayesian meta-regression | Residual between-arm SD | 1.006 | 17,800 |

R-hat values close to 1.00 and high effective sample sizes supported adequate convergence of the full MCMC models. SD, standard deviation.
